# Introduction to the Special Issue “Molecular Basis and the Pathogenesis of Enterohemorrhagic *Escherichia coli* Infections”

**DOI:** 10.3390/toxins12120763

**Published:** 2020-12-03

**Authors:** Kim Stanford

**Affiliations:** Research and Innovation Services, University of Lethbridge, 4401 University Drive, Lethbridge, AB T1K 3M4, Canada; kim.stanford@uleth.ca

Although much of the world has progressed since the 1980s, our ability to treat infections with enterohemorrhagic *Escherichia coli* (EHEC) has unfortunately shown little improvement. This Special Issue is a collection of 10 articles that provides new information which challenges old beliefs about EHEC colonization, virulence, pathogenicity, mitigation, and surveillance. As EHEC control involves a One Health approach, including both agriculture and human medicine that encompasses food supply chains and is directed by effective surveillance strategies, this Special Issue includes examples of innovation in each sector and outlines the steps required to reduce future EHEC human health risks.

In contrast to an earlier belief that cattle resisted detrimental effects of Shiga toxins, a comprehensive review of the literature demonstrates that EHEC plays a pivotal role in cattle colonization, with implications for future improved EHEC controls to prevent human disease [1]. Strains of EHEC that sporadically or persistently colonize the gastrointestinal tract of cattle were shown to differ. Sporadic colonizers conserved features that promote their survival in the environment, while persistent strains may have a greater risk for human disease, as they carry genetic mutations that could facilitate their future detection [2]. Potential new avenues for EHEC control were further developed in a review of current knowledge of the Shiga toxin and cell interactions at the molecular level [3], including Shiga toxin modulation of intercellular communication, a key component of EHEC pathogenesis. Future treatments for hemolytic uremic syndrome (HUS) would combat bacterial virulence factors instead of passively treating kidney failure, as discussed in a review of mechanisms of EHEC virulence [4]. The lack of progress in EHEC treatments was illustrated from work showing a decline in EHEC infections since the 1980s in the Province of Alberta, Canada, even though HUS still occurs in 5% of infections [5]. This lack of change in HUS incidence over time may also be influenced by changes in dominant EHEC strains. Strains of EHEC with a Shiga toxin subtype stx2a were found to cause an increased risk of HUS compared to those carrying both stx2a and stx1a [6]. Mechanisms for the interaction between stx1a and stx2a to attenuate toxicity have not been determined, but may be a fruitful approach to mitigate EHEC pathogenesis. Another potential new avenue to control EHEC would be the use of bacteriocins, although the bacteriocin must first be separated from its immunity gene(s) for efficacy [7]. A novel mechanism, where the Shiga toxin 1B subunit is sequestered in extracellular vesicles derived from blood cells, demonstrates how the toxin may evade host immune responses [8], providing a better understanding of the mechanism for EHEC pathogenesis. How better to control EHEC in lettuce was shown by the increasing tolerance of EHEC to chlorine the longer it was present on the lettuce [9]. Finally, the need for effective surveillance of EHEC to be tailored to local needs was demonstrated by an eight-year survey in South Africa [10]. In South Africa, O26:H11 was the most common serotype that caused human disease, followed by O111:H8. O157:H7 was in third place, and tied with O117:H7, a serotype overlooked in many jurisdictions.
